# Determinants of substantial public debt reductions in Central and Eastern European Countries

**DOI:** 10.1007/s10663-021-09529-2

**Published:** 2021-11-22

**Authors:** Sofia Semik, Lilli Zimmermann

**Affiliations:** 1 Goethe University, 60323 Frankfurt am Main, Germany; 2 Deutsche Bundesbank, University of Applied Sciences, Schloss, 57627 Hachenburg, Germany

**Keywords:** Public debt, Fiscal policy, Central and Eastern Europe, Logistic probability model, C35, E62, H6

## Abstract

Government debt development is a timeless issue in economics that has gained even more attention in light of the global financial crisis and the Covid 19 pandemic crisis. The following paper uses several specifications of a logistic probability model to examine the key determinants underlying substantial public debt reductions in Central and Eastern European EU Member States for the period 1996–2020. The results suggest that fiscal adjustments are more likely to be successful in reducing public debt if they are based on expenditure cuts rather than revenue increases. In this context, cuts in social benefits and government employee compensation prove to be particularly effective. In addition, favourable economic growth rates increase the probability of a substantial reduction in government debt.

## Introduction

The global financial crisis of 2007/08 has led to unprecedentedly high levels of public debt in many countries around the world. In this context, recent developments related to the outbreak of the Covid-19 pandemic will pose additional challenges due to substantial government support to firms and households as well as extreme losses in output (IMF 2020). The EU’s average gross general government debt-to-GDP ratio has risen significantly, from 58 percent in 2007 to over 90 percent in 2020. The public debt levels of many countries are expected to increase further in 2021 owing to the rescue packages in course of the pandemic crisis.

Although the eleven Central and Eastern European EU member states (CEECs) are on average much less indebted than more advanced EU members, the increased public debt still poses considerable risks for these countries. The average gross general government debt-to-GDP ratio in these countries has increased from 26 percent in 2007 to 54 percent in 2020. As the market tolerance towards public debt is generally lower in emerging market economies, the CEECs are not spared the risks of rising and persistently high government debt levels (Darvas 2010). Furthermore, six of the CEECs still need to fulfil the Maastricht criteria, which contain thresholds for the government deficit and debt level, in order to join the euro area.

This paper aims to identify the key factors that have led to substantial and long-lasting reductions of public debt in the CEECs in the past in order to draw possible conclusions about promising measures for the future. Using data for eleven CEECs from 1996 to 2020, several logistic probability models are estimated to assess which factors contributed to substantial reductions in the gross general government debt-to-GDP ratio during the period concerned.

The remainder of the paper is structured as follows. In Sect. 2 related literature on government debt reduction is discussed. Section 3 provides an overview of debt developments in the CEECs and identifies episodes of substantial public debt reduction within the data set. In Sect. 4, the main determinants of public debt dynamics are examined. The empirical framework is set out in Sect. 5, followed by the presentation of the estimation results. The conclusion is then drawn in Sect. 6.

## Literature review

The issue of successfully reducing public debt has become increasingly important since the sharp rise in public debt ratios in the wake of the global financial crisis in 2008/09. Various studies examine the driving forces behind earlier public debt reductions with the aim of identifying promising policy measures (Baldacci and Kumar 2010; Boussard et al. 2012; Baldacci et al. 2013; Akitoby et al. 2014; Eyraud and Weber 2013; Bellettini and Roberti 2020). Our analysis follows the approach of Nickel et al. (2010). They estimate a logistic probability model to examine which factors contributed to major reductions in the government debt-to-GDP ratio in the EU15 countries from 1985 to 2009. A major debt reduction in this context is defined as a decline in the debt ratio by at least ten percentage points over five consecutive years. Their results suggest that persistent fiscal consolidations that are mainly based on expenditure cuts, especially on cuts in social benefits and public wages, support major debt reductions. Moreover, they note that high real trend GDP growth and a high interest burden on the government increases the likelihood of a substantial decline in the public debt ratio.

Cherif and Hasanov (2012) apply a vector-autoregression (VAR) model with debt feedback in order to examine the impulse response of the public debt-to-GDP ratio following fiscal, growth and inflation shocks using data from the US. They find that fiscal adjustments and improvements in economic growth ensure the reduction of government debt over the medium term. Inflation shocks, however, are not found to be a suitable instrument to support debt reduction sustainably. In line with these findings, the results of Abbas et al. (2013) suggest that the structural primary budget balance and economic growth are the key determinants of large previous public debt reductions. Here a large debt reduction is defined as a decline in the gross public debt-to-GDP ratio of at least five percentage points over four consecutive years. They use standard debt dynamics calculations with the aim of identifying appropriate policy measures for successful government debt reduction in a macroeconomic environment of low growth. Alesina et al. (2019) examine the effectiveness of expenditure- and tax-based fiscal austerity plans using data from 16 OECD countries. They use a vector autoregression which includes taxes, government expenditure, interest expenses on government debt, real GDP growth and inflation and find that expenditure-based austerity plans are more effective in reducing public debt significantly, especially in the long run. Their results further suggest that tax-based fiscal austerity may have debt-increasing effects in the short run due to its contractionary effect on the economy. In this context, several studies find that permanent and mainly expenditure-based fiscal adjustments are more likely to lead to a successful and sustained reduction in government debt (Alesina and Perotti 1996; Giavazzi et al. 2000; Alesina and Ardagna 2013; Alesina et al. 2015).

Up to now, research on the determinants of public debt reductions has mainly focused on advanced economies in Europe and the US. The limited attention for debt reduction strategies in CEECs is, however, unfounded given sizeable debt-to-GDP ratios in some of these countries and the broad agreement on the negative economic effects of persistently high public debt ratios, especially in emerging economies.

Afonso et al. (2006) estimate a logistic probability model to examine the factors underlying successful fiscal contractions, defined as a sizeable and lasting improvement in the primary budget balance, in ten CEECs over the period 1991–2003. They find that consolidations based on expenditure cuts are likely to contribute to a successful fiscal adjustment while revenue-based consolidations tend to decrease the probability of success. Cuestas (2020) uses a debt sustainability analysis to assess the changes in public debt dynamics in eleven CEECs since the strong accumulation of debt in the wake of the crisis in 2008/09. His findings show that most of the countries have succeeded in putting their public debt back on a sustainable path since 2009. While Cuestas focuses solely on public debt dynamics, our study examines the individual factors that influence the success of debt reductions.

## Public debt developments in the CEECs

### Evolution of government debt-to-GDP ratios in the CEECs

While a lot of research has been made analysing public debt reductions in the US and the core European countries, there is a lack of literature analysing the determinants of persistent and long-lasting debt reductions in Central and Eastern Europe. Therefore, we analyse the debt developments of eleven Central and Eastern European EU member states, that joined the European Union in 2004, 2007 and 2013.^1^ Table 1 shows the evolution of gross general government debt-to-GDP ratios in the CEECs.

**Table 1:** Gross general government debt-to-GDP ratios in the CEECs. *Source* European commission, AMECO database; IMF world economic outlook database; IMF: A historical public debt database

	1995	2000	2005	2010	2015	2019	2020
Bulgaria	104.33	70.66	26.58	15.39	25.99	21.14	25.72
Croatia	27.92	35.51	41.30	57.74	84.43	71.17	82.34
Czech Republic	13.66	17.04	27.88	37.35	39.96	31.47	37.89
Estonia	7.96	5.11	4.70	6.61	10.00	8.65	22.52
Hungary	84.04	55.71	60.64	80.63	76.14	68.19	77.92
Latvia	13.93	12.11	11.42	47.27	36.65	35.98	45.92
Lithuania	11.52	23.49	17.63	36.31	42.71	36.26	50.66
Poland	47.57	36.45	46.44	53.13	51.29	47.42	57.29
Romania	6.61	22.49	15.91	29.63	37.77	35.47	54.60
Slovakia	21.58	50.45	34.73	40.99	51.89	48.12	65.68
Slovenia	18.24	25.92	26.40	38.27	82.59	66.67	80.17

Apart from the strong heterogeneity of public debt developments in the CEECs, the debt-to-GDP ratios show on average a declining trend from 1995 to 2005. This may reflect the increasingly favourable macroeconomic environment within these countries at that time, driven by the transition to a market economy in the 1990s and the accession to EU (ECB 2004). The impact of the financial and economic crisis of 2008/2009 is clearly reflected in the rising debt ratios of the CEECs from 2005 to 2015. In order to restore the economic activity that collapsed in the wake of the crisis, many governments, including those of the CEECs, pursued discretionary expansionary fiscal policies and allowed automatic stabilizers to work (Cuestas 2020). Although all the CEECs managed to decrease their government debt ratio since 2015, most countries are still far above their pre-crisis levels of 2005. In 2019, the public debt ratio of Croatia, Hungary and Slovenia even exceeds the threshold of 60 percent of GDP set by the Maastricht Treaty. Despite the relatively low debt levels compared to the EU15 countries,^2^ the CEECs are strongly affected by rising debt levels due to lower debt tolerance for emerging market economies (Darvas 2010).

### Identification of substantial public debt reduction episodes

We define a substantial public debt reduction as a decline in the gross general government debt-to-GDP ratio by more than six percentage points over five consecutive years, cumulatively. To account for lower overall public debt levels in the CEECs, if compared to more advanced economies in Europe, we apply a lower threshold in comparison to previous studies.^3^ The threshold of six percentage points ensures that substantial debt reductions are separated from moderate ones in this region. In addition, the condition of five consecutive years allows us to avoid one-off effects.

Our sample covers annual data on changes in the gross general government debt-to-GDP ratio for the eleven CEECs for the period 1996–2020, containing a total of 275 observations. Within the sample 144 observations can be defined as debt accumulation episodes with a positive annual change in the gross general government debt-to-GDP ratio. The remaining 131 episodes are considered debt reduction episodes, distinguishing between 31 substantial debt reductions, according to our definition, and 100 moderate debt reduction episodes. The following table lists all periods in which substantial debt reductions occurred within the CEECs during the period under consideration (Table 2).

**Table 2:** Periods of substantial public debt reduction. *Source* European commission AMECO database; IMF world economic outlook database; IMF: A historical public debt database; own calculations

Country	Period of substantial debt reduction	Public debt ratio (in % of GDP)		Change in debt ratio
	[t_0_–t_n_]	Peak [t_−1_]	Trough [t_n_]	
Bulgaria	1997–2008	141.31	13.02	− 128.29
Croatia	2015–2019	84.68	71.17	− 13.51
Czech Republic	2014–2019	44.91	31.47	− 13.44
Estonia				
Hungary	1996–2001	84.04	52.25	− 31.79
	2012–2019	80.80	68.19	− 12.61
Latvia				
Lithuania	2001–2008	23.49	14.56	− 8.93
Poland	1996–2000	47.57	36.45	− 11.12
Romania	2002–2007	25.87	11.95	− 13.92
Slovakia	2002–2008	51.11	28.60	− 22.51
Slovenia				

All CEECs, except for Estonia, Latvia, and Slovenia, experienced at least one substantial debt reduction period. Hungary experienced even two periods of substantial debt reductions during the time span 1996–2020. This outcome is not surprising for Estonia, as the country has a very low debt ratio throughout the period considered, peaking at 10.55 percent of GDP in 2014. This makes a large reduction by 6 percentage points highly unrealistic. Among the countries that experienced a substantial debt reduction, Bulgaria shows an outstanding performance given the most persistent debt reduction period of eleven years and an overall debt decline of almost 130 percentage points In comparison, the average substantial public debt reduction in the sample amounts to somewhat 30 percentage points. Excluding Bulgaria’s outlier value, the average debt reduction amounts to about 16 percentage points. While most episodes of substantial debt reductions took place before the outbreak of the financial crisis in 2008, there have been substantial debt reductions in recent years in Croatia, Hungary and the Czech Republic.

## Public debt dynamics

Changes in the gross government debt-to-GDP ratio can be decomposed as follows:

1 $$\Delta {\text{d}}_{{\text{t}}} = \frac{{{\text{r}}_{{\text{t}}} - {\text{g}}_{{\text{t}}} }}{{1 + {\text{g}}_{{\text{t}}} }} * {\text{d}}_{{{\text{t}} - 1}} - {\text{pb}}_{{\text{t}}} + {\text{dda}}_{{\text{t}}}$$

The first component $\frac{{{\text{r}}_{{\text{t}}} - {\text{g}}_{{\text{t}}} }}{{1 + {\text{g}}_{{\text{t}}} }} * {\text{d}}_{{{\text{t}} - 1}}$ represents the interest rate growth differential and is known as the snowball-effect. According to the snowball-effect, the debt-to-GDP ratio tends to increase if the GDP growth rate is lower than the interest rate paid on public debt. The second component $- {\text{pb}}_{{\text{t}}}$ represents the primary balance, which in case of a deficit tends to increase public debt.

The third component (${\text{dda}}_{{\text{t}}}$) deficit-debt adjustment reflects differences between the change in government debt and the change in government deficit. These may, for instance, arise from the purchase of assets, from shifts in the value of foreign currency-denominated debt as a result of fluctuations in exchange rates, from government support to private financial institutions or from privatization revenues. However, the debt-relief effect of such adjustments is rather small, as for example privatizations or the sale of financial assets cannot generate unlimited revenue for the government, especially in a weak economic environment (ECB 2012).

**Fig. 1:**
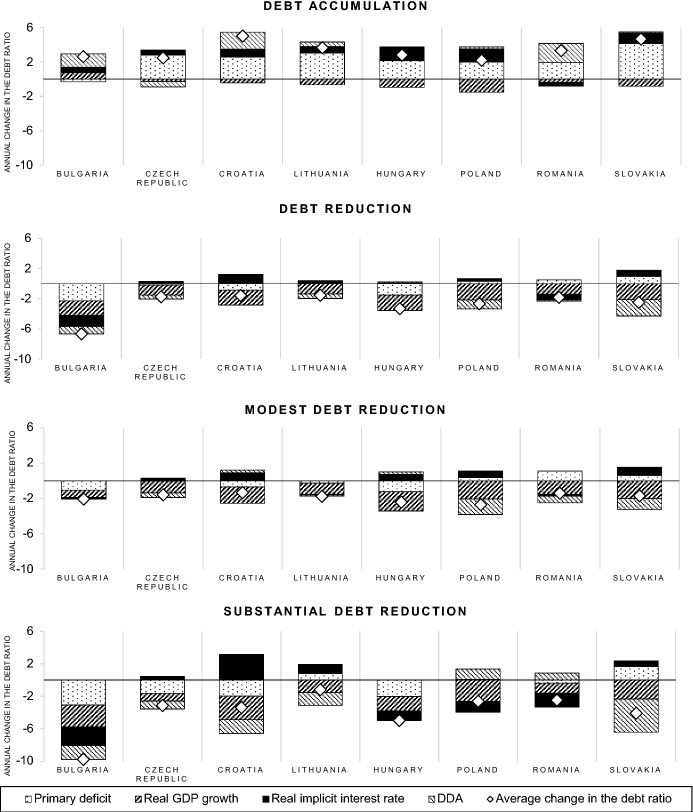
Contributing factors to changes in the public debt-to-GDP ratio. *Notes* The real GDP growth is included with a reversed sign in order to illustrate the positive contribution to reductions of the public debt-to-GDP ratio. The data on real interest rates refer to the implicit interest rate, i.e. the general government’s interest expenditure as a percentage of gross public debt of the preceding year, adjusted for inflation (GDP deflator) *Source* European commission AMECO database; IMF world economic outlook database; own calculations

The key determinants of changes in the public debt ratio, according to Eq. 1, are the real interest rate governments pay on their outstanding debt (${\text{r}}_{{\text{t}}}$), the real GDP growth rate (${\text{g}}_{{\text{t}}}$), the primary budget balance and the deficit-debt adjustment. Figure 1 illustrates how these determinants contributed to changes in the public debt ratio of the CEECs who experienced substantial debt reductions during the period under consideration. It considers the contribution of the factors during episodes of debt accumulation and debt reduction, distinguishing between modest and substantial debt reduction episodes.

During the period 1996–2020 the CEECs accumulated public debt predominantly because of high primary deficits. The main driver of debt reductions has been high real GDP growth. In addition, the real implicit interest rate has been significantly lower in most CEECs during episodes of debt reduction. This might reflect the increasing creditworthiness of governments in times of fiscal consolidation. The deficit-debt adjustment contributed only marginally to modest and substantial debt reductions in most CEECs. An exceptionally large contribution of deficit-debt adjustments to substantial debt reductions can be observed in the case of Slovakia. This high value is mainly attributable to large privatization proceeds in 2002.

The average government debt-to-GDP ratio decline during the episodes of substantial debt reductions amount to 6.5 percent, while the average decline during the modest debt reduction episodes only amounts to about 2 percent. Although economic growth appears to be the main driver of modest and substantial debt reductions, the primary surpluses are significantly higher, with 1.3 percent of GDP on average, during the substantial debt reduction episodes. In contrast, during periods of modest debt reductions the average primary surplus amounts to 0.4 percent of GDP. The contribution of the real implicit interest rate to the success of debt reduction cannot be derived conclusively from Fig. 1. On the one hand, low interest rates contribute to a reduction of the public debt ratio, as can be inferred from Eq. 1. On the other hand, sovereign bond yields tend to increase in response to high public debt levels in order to compensate for the higher estimated risk of default. Since high debt servicing costs accelerate debt accumulation, reducing debt would become increasingly difficult. High interest rates could therefore play a disciplinary role for public debt reduction as they put additional pressure on the government to reduce its debt (Nickel et al. 2010).

## Empirical analysis

### Methodology

In this section we empirically examine the determinants of significant public debt reductions in the CEECs for the period 1996–2020. Using annual data and different specifications of a logistic probability model, we assess which of the factors discussed in Sect. 4 rather contribute to significant and long-lasting public debt reductions in the countries under consideration. The analysis is based on a truncated panel covering 131 debt reduction episodes. According to our definition of a successful public debt reduction, our sample consists of 31 successful episodes and 100 unsuccessful episodes. The logistic probability model is defined as follows:

2 $${\text{P}}_{{\text{i}}} = {\text{E(S}} = 1{\text{ |Z}}_{{\text{i}}} ) = \frac{{{\text{e}}^{{{\text{Z}}_{{\text{i}}} }} }}{{1 + {\text{e}}^{{{\text{Z}}_{{\text{i}}} }} }}$$

${\text{P}}_{{\text{i}}}$ represents the conditional probability that a substantial and, therefore, successful debt reduction occurs, given ${\text{Z}}_{{\text{i}}}$. ${\text{E(S}} = 1{\text{ |Z}}_{{\text{i}}} )$ denotes the conditional expectation of the success of a public debt reduction. The binary variable S is defined in the following way:

3 $${\text{S}} = \left\{ \begin{gathered} 1,\;{\text{in case~}}\;{\text{of~}}\;{\text{a~}}\;{\text{substantial}}\;{\text{debt~}}\;{\text{reduction}} \hfill \\ 0,\;{\text{in case}}\;{\text{of}}\;{\text{a}}\;{\text{modest~}}\;{\text{debt~}}\;{\text{reuction}} \hfill \\ \end{gathered} \right.$$

The logistic regression model is based on the variable ${\text{Z}}_{{\text{i}}}$, which is defined as follows:

4 $${\text{Z}}_{{\text{i}}} = \log \left( {\frac{{{\text{P}}_{{\text{i}}} }}{{1 - {\text{P}}_{{\text{i}}} }}} \right) = \beta_{0} + \beta_{1} {\text{fi}}_{{\text{i}}} + \beta_{2} {\text{PEXP}}_{{\text{i}}} + \beta_{3} {\text{trendgr}}_{{\text{i}}} + \beta_{4} {\text{gap}}_{{\text{i}}} + \beta_{5} {\text{interest}}_{{\text{i}}}$$

The definition of the conditional probability in Eq. 4 (${\text{P}}_{{\text{i}}} = \frac{{{\text{e}}^{{{\text{Z}}_{{\text{i}}} }} }}{{1 + {\text{e}}^{{{\text{Z}}_{{\text{i}}} }} }}$) allows to bound the results of a logistic regression to values between 0 and 1. Whereas β_0_ represents the constant term. The variable ${\text{fi}}_{{\text{i}}}$ represents the fiscal impulse derived from the general government primary budget balance in the year prior to the debt reduction period. This controls for the effect that debt reductions are more likely to be successful starting from high primary balances (Giavazzi 2000). In order to control for the composition of fiscal consolidations in order to examine whether expenditure cuts are more effective in terms of debt reduction than increases in the government’s revenue, we include a dummy variable ${\text{PEXP}}_{{\text{i}}}$. That variable reflects whether the change in the primary expenditure ratio is significant towards the total change in the primary balance as a percentage of GDP. The construction of this dummy variable is displayed in Eq. 5.

5 $${\text{PEXP}}_{{\text{t}}} = \left\{ \begin{gathered} 1,~if~\left( {\frac{{\Delta {\text{PEXP}}_{{\text{t}}} }}{{\Delta {\text{pb}}_{{\text{t}}} }}} \right) > \lambda \hfill \\ 0,~~otherwise \hfill \\ \end{gathered} \right.$$

Accordingly, the dummy variable takes the value of one if current cuts in the primary expenditure ratio account for at least $\lambda$ percent of the overall change in the primary budget balance. In this context, “current cuts in primary expenditure” refer to changes in the ratio of primary government expenditure to the GDP of the respective country.

The remaining three explanatory variables reflect the effects of the current macroeconomic environment and the government’s interest burden on the likelihood of successful public debt reductions. The variable ${\text{trendgr}}_{{\text{i}}}$ represents the real trend growth of the economy. It is computed by applying the Hodrick-Prescott-Filter (HP-Filter) on the real GDP growth series for each CEEC. We use the HP filter following the argumentation of Schueler (2018) and Hodrick (2020). Both recommend the HP filter as the most appropriate for determining the trend component of real GDP growth by extracting business cycle fluctuations. In addition, the change in the output gap (${\text{gap}}_{{\text{i}}}$) is included. It is computed as the difference between real GDP growth and real GDP trend growth. The impact of the current interest burden of the government will be assessed using the explanatory variable ${\text{interest}}_{{\text{i}}}$. It represents the government’s debt financing costs as a percentage of GDP.

For robustness reasons we also assess an alternative definition of the variable ${\text{Z}}_{{\text{i}}}$ with respect to the fiscal adjustment composition dummy variable.

6 $${\text{Z}}_{{\text{i}}} = \beta_{0} + \beta_{1} {\text{fi}}_{{\text{i}}} + \beta_{2} {\text{REV}}_{{\text{i}}} + \beta_{3} {\text{trendgr}}_{{\text{i}}} + \beta_{4} {\text{outputg}}_{{\text{i}}} + \beta_{5} {\text{interest}}_{{\text{i}}}$$

The primary expenditure dummy is thereby replaced by a revenue dummy.

7 $${\text{REV}}_{{\text{t}}} = \left\{ \begin{gathered} 1,~if~\left( {\frac{{\Delta {\text{REV}}_{{\text{t}}} }}{{\Delta {\text{pb}}_{{\text{t}}} }}} \right) > \lambda \hfill \\ 0,~~otherwise \hfill \\ \end{gathered} \right.$$

The revenue dummy takes the value of one if current increases in the revenue ratio of the government account for at least $\lambda$ percent of the overall change in the primary balance.

### Estimation results

#### Determinants of substantial public debt reductions

The estimation results of the logistic regression, based on Eqs. 4 and 6 respectively, are shown in Table 3.

**Table 3:** Estimation results

	Threshold λ = 80		Threshold λ = 60	
	(1)	(2)	(1)	(2)
Fiscal impulse	0.38* (0.22)	0.38 (0.23)	0.36 (0.23)	0.30 (0.27)
PEXP	1.13** (0.53)		0.83* (0.47)	
REV		− 0.32 (0.64)		− 0.30 (0.59)
Trend growth	0.55** (0.28)	0.53** (0.27)	0.54** (0.28)	0.53** (0.26)
Output gap	0.05 (0.09)	0.07 (0.09)	0.05 (0.09)	0.07 (0.08)
Interest burden	0.81** (0.36)	0.76** (0.38)	0.79* (0.40)	0.77* (0.45)
Constant	− 5.52*** (1.35)	− 4.93*** (1.40)	− 5.36*** (1.50)	− 4.94*** (1.38)
Observations	131	131	131	131
Substantial debt reductions (S = 1)	31	31	31	31
McFadden R^2^	0.26	0.23	0.25	0.23
Wald χ^2^ (5) statistics	17.94	8.95	9.19	9.47
Marginal effects				
Fiscal impulse	0.04	0.04	0.04	0.04
PEXP	0.13		0.10	
REV		− 0.04		− 0.04
Trend growth	0.07	0.07	0.07	0.07
Output gap	0.01	0.01	0.01	0.01
Interest burden	0.09	0.10	0.10	0.10

Cluster robust standard errors are given in parenthesis. ***, **, * Refer to statistical significance at 1%, 5% and 10% levels, respectively

In case of dummy variables the marginal effects refer to the discrete change from 0 to 1

The threshold for the primary expenditure and revenue dummy variables ($\lambda$) is set once at 80 percent and once at 60 percent of the overall change in the primary budget balance to ensure the robustness of the outcome. In order to take account of the potential heterogeneity between different debt reduction periods, the estimation is based on cluster-robust standard errors. This allows the episodes to be uncorrelated across clusters but to be correlated within clusters. This is crucial as the episodes of public debt reductions under consideration occurred consecutively over longer time periods. As the impact of the explanatory variables on the probability of a substantial debt reduction cannot be derived directly from the level of the coefficient in a binary model, the average marginal effect (${\text{dP}}/{\text{dZ}}$) for each independent variable is displayed in Table 3. The average marginal effects show how a one-unit change in the average value of the independent variables affects the probability of a substantial public debt reduction and thus enable a more conclusive interpretation of the estimation results.

The control variable for the fiscal impulse has the expected positive sign, suggesting that high primary surpluses facilitate substantial public debt reductions. However, it turns out to be insignificant for three out of four model specifications. The composition of the fiscal adjustment appears to be a striking factor in determining the likelihood of a successful government debt reduction. According to the estimation results, primary expenditure cuts significantly contribute to successful debt reductions. The dummy variable has the expected positive sign and is statistically significant. This reflects that public debt reductions are more likely to be substantial if current expenditure cuts account for at least 80 or 60 percent, respectively, of the fiscal tightening. As can be seen from the marginal effects, a change in the dummy variable from zero to one increases the probability of a substantial debt reduction by 13 percent in case of the 80 percent threshold. On the other hand, the revenue dummy has a negative sign and is statistically insignificant. Hence, revenue-based fiscal consolidations do not seem to contribute positively to the probability of substantial public debt reductions.

Furthermore, the results suggest that real trend growth is a determining factor for the success of a debt reduction. A one percent increase in the trend growth raises the probability of a substantial public debt reduction by 7 percent. Therefore, the implementation of structural reforms that support GDP trend growth appears necessary to reduce debt successfully, especially during economic downturns (Abbas et al. 2013). The impact of the real output gap, however, turns out to be statistically insignificant. This indicates that positive changes in the output gap, which are associated with the economy operating above its potential and thus rising inflationary pressures, did not induce strong fiscal tightening and therefore had no significant impact on the success of government debt reductions.

In addition, the government’s interest burden seems to have a significant impact on the probability of major public debt reductions. The estimation results suggest that a one percent increase in the interest burden increases the probability by 9 percent and that the coefficient is statistically significant. High public debt levels tend to boost sovereign risk premia, and thereby interest rates, due to a higher estimated default risk of the government. Thus, it can be inferred that increasing debt servicing costs represent an incentive for governments to reduce their debt substantially.

The constant term turns out to be negative and statistically significant. This could be the result of deficit and debt biases in the economy. These biases could be the result of political dynamics, such as upcoming elections and insufficiently informed voters. For instance, policy makers may tend to increase government spending rather than taxes before elections or during a recession in order to appease their voters which leads to biased deficits and debt ratios (Schuknecht 2004; Price 2010).

#### Successful government expenditure cuts

As fiscal adjustments based on expenditure cuts seem to contribute significantly to successful public debt reductions, the following section analyzes which position of government spending has been especially effective in the CEECs in terms of debt reduction. The assessment includes five positions of general government expenditure: the compensation of employees, social benefits, final government consumption of goods and services, gross fixed capital formation and subsidies.

For the evaluation of the effectiveness of these components of primary expenditure cuts, a slightly different version of the logistic regression described in Sect. 5.2.1 is estimated. We use a modified definition of the variable ${\text{Z}}_{{\text{i}}}$ as shown in the following equation.

8 $${\text{Z}}_{{\text{i}}} = \beta_{0} + \beta_{1} {\text{fi}}_{{\text{i}}} + \beta_{2} {\text{trendgr}}_{{\text{i}}} + \beta_{3} {\text{outputg}}_{{\text{i}}} + \beta_{4} {\text{interest}}_{{\text{i}}} + \beta_{5} {\text{component}}_{{\text{i}}}$$

The variable ${\text{component}}_{{\text{i}}}$ is a dummy variable which controls for the composition of the cut in government expenditure that leads to a fiscal adjustment. It thereby reflects if the change in the respective component is significant vis-à-vis the overall change in the primary expenditure ratio. The dummy variable is constructed as follows:

9 $${\text{component}}_{{\text{t}}} = \left\{ \begin{gathered} 1,\,~if~\left( {\frac{{\Delta {\text{component}}_{{\text{t}}} }}{{\Delta {\text{PEXP}}_{{\text{t}}} }}} \right) > \mu \hfill \\ 0,~~otherwise \hfill \\ \end{gathered} \right.$$

Accordingly, the variable takes the value of one if the share of the reduction in the respective component of the overall change in the primary general government expenditure ratio is higher than its average value among all expenditure-based fiscal consolidations. For the purpose of this estimation, a fiscal consolidation is defined as expenditure-based if current primary expenditure cuts account for at least 60 percent of the change in the primary balance ($\lambda$ = 60). Table 4 presents the estimation results of Eq. 8.

**Table 4:** Estimation results–expenditure composition

	(1)	(2)	(3)	(4)	(5)
Fiscal impulse	0.36* (0.22)	0.38 (0.25)	0.33 (0.23)	0.33 (0.23)	0.31 (0.23)
Trend growth	0.52** (0.26)	0.54** (0.27)	0.53** (0.27)	0.56** (0.27)	0.55** (0.27)
Output gap	0.04 (0.09)	0.04 (0.09)	0.05 (0.09)	0.06 (0.09)	0.07 (0.09)
Interest burden	0.76** (0.36)	0.77** (0.38)	0.77** (0.37)	0.78** (0.37)	0.78** (0.38)
Compensation of employees	0.92** (0.43)				
Social benefits		1.17* (0.60)			
Government consumption			0.53 (0.45)		
Gross fixed capital formation				0.66 (0.48)	
Subsidies					0.40 (1.04)
Constant	− 5.11*** (1.35)	− 5.28*** (1.33)	− 5.10*** (1.38)	− 5.17*** (1.40)	− 5.08*** (1.40)
Observations	131	131	131	131	131
Major debt reductions (S = 1)	31	31	31	31	31
McFadden R^2^	0.25	0.26	0.24	0.24	0.23
Wald χ^2^ (5) statistics	14.61	13.38	11.22	10.55	9.34
Marginal effects					
Fiscal impulse	0.05	0.05	0.04	0.04	0.04
Trend growth	0.07	0.07	0.07	0.07	0.07
Output gap	0.01	0.01	0.01	0.01	0.01
Interest burden	0.10	0.09	0.10	0.10	0.10
Compensation of employees	0.12				
Social benefits		0.14			
Government consumption			0.07		
Gross fixed capital formation				0.09	
Subsidies					0.05

Cluster robust standard errors are given in parenthesis. ***, **, *Refer to statistical significance at 1%, 5% and 10% levels, respectively

In case of dummy variables the marginal effects refer to the discrete change from 0 to 1

The results suggest that primary expenditure cuts based on reductions in employee compensation and social benefits have a significant impact on the likelihood of substantial public debt reductions. If the cut in government employee compensation is significant relative to the overall decline in the primary expenditure ratio, the probability of a substantial debt reduction increases by 12 percent. In case of a significant decline in social benefits, the marginal effect on the probability amounts to 14 percent. On the other hand, the impact of government spending cuts based strongly on reductions in government consumption expenditure, gross fixed capital formation or subsidies on the success of public debt reductions is statistically insignificant.

### Robustness tests

In order to assess the robustness of the estimated results, three alternative specifications of the logistic probability model are performed.

First, an alternative definition of a substantial debt reduction period is applied. As the threshold of at least 6 percentage points is relatively low if compared to previous studies on successful debt reductions, we run an estimation applying a more demanding threshold, defining a substantial debt reduction as a decline in the gross general government debt-to-GDP ratio by more than ten percentage points over five consecutive years. Consequently, the number of substantial debt reductions within the truncated data panel of 131 observations decreases from 31 to 21 episodes. Table 5 in the appendix shows the estimation results for the alternative definition of a substantial debt reduction period. The results do not differ significantly from the results applying the 6 percent limit.

The second modification accounts for potential country-specific effects among the eleven CEECs included in the sample. Since panel heterogeneity across the countries could result in within-cluster autocorrelation in the error term, this estimation is based on cluster-robust standard errors in order to account for potential heterogeneity between the different countries under review. This allows for the specification that observations are uncorrelated across countries but may be correlated within countries. Table 6 in the appendix shows that the results of this modified estimation are consistent with those of the baseline estimation.

Finally, we consider a specification in which every second observation was omitted for each individual country. This ensures a higher degree of independence of the variables since the observations in the baseline estimation might be correlated. As can be seen in Table 7 in the appendix, the results of this modification are not significantly different from the original sample.

## Conclusion

The successful reduction of government debt has proven to be an important issue, especially since the global financial crisis in 2008. Moreover, it becomes all the more topical in the light of recent economic developments related to the outbreak of the Covid-19 pandemic as it implies additional challenges to the maintenance of sound public finances (IMF 2020). Therefore, this paper aims to identify the main determinants underlying past substantial public debt reductions, using data from eleven CEECs in the period 1996–2020. The results, which are derived from the estimation of different specifications of a logistic probability model, underline the importance of fiscal policy composition, economic growth as well as the interest burden on the government for substantial reductions in the public debt-to-GDP ratio.

In terms of fiscal policy, fiscal adjustments are found to be most successful when they are mainly based on cuts in government expenditure. In this context, reducing government employee compensation and social benefit payments seem to be the most effective instruments for substantial public debt reductions. On the other hand, according to the results, fiscal consolidation efforts that are focussed on increasing government revenues are apparently not contributing to the success of debt reductions. Furthermore, the results suggest that high rates of real GDP trend growth significantly increase the probability of substantial public debt reductions. Therefore, the implementation of structural reforms to improve trend growth is essential for significantly reducing government debt (Abbas et al. 2013). Finally, the interest burden on the government appears to be an important factor underlying substantial debt reductions. If interest rates rise in line with the public debt ratio due to credibility concerns, the government may find itself forced to reduce its debt in order to prevent the acceleration of debt accumulation as a result of increasing debt service costs. Further research is, however, required to shed more light on this issue.

## Appendix

See Tables 5, 6 and 7.

**Table 5:** Estimation results–alternative debt threshold

	Threshold λ = 80		Threshold λ = 60	
	(1)	(2)	(1)	(2)
Fiscal impulse	0.50 (0.35)	0.44 (0.36)	0.50 (0.38)	0.42 (0.35)
PEXP	1.15*** (0.40)		0.98** (0.41)	
REV		0.46 (0.77)		− 0.08 (0.87)
Trend growth	0.46** (0.20)	0.45** (0.27)	0.44** (0.20)	0.42** (0.26)
Output gap	0.10 (0.10)	0.11 (0.10)	0.10 (0.10)	0.11 (0.09)
Interest burden	0.83*** (0.27)	0.78*** (0.38)	0.79** (0.32)	0.77*** (0.27)
Constant	− 6.08*** (1.22)	− 5.63*** (1.41)	− 5.95*** (1.45)	− 5.43*** (1.39)
Observations	131	131	131	131
Major debt reductions (S = 1)	21	21	21	21
McFadden R^2^	0.32	0.30	0.31	0.29
Wald χ^2^ (5) statistics	36.97	14.96	13.63	18.57
Marginal effects				
Fiscal impulse	0.01	0.01	0.01	0.01
PEXP	0.03		0.03	
REV		0.01		− 0.003
Trend growth	0.01	0.01	0.01	0.01
Output gap	0.003	0.003	0.003	0.004
Interest burden	0.02	0.02	0.02	0.03

Cluster robust standard errors are given in parenthesis. ***, **, *Refer to statistical significance at 1%, 5% and 10% levels, respectively

In case of dummy variables the marginal effects refer to the discrete change from 0 to 1

**Table 6:** Estimation results–panel heterogeneity clusters

	Threshold λ = 80		Threshold λ = 60	
	(1)	(2)	(1)	(2)
Fiscal impulse	0.38 (0.26)	0.30 (0.27)	0.36 (0.28)	0.30 (0.27)
PEXP	1.13** (0.54)		0.85 (0.54)	
REV		− 0.32 (0.67)		− 0.30 (0.59)
Trend growth	0.56* (0.29)	0.53** (0.26)	0.54* (0.29)	0.53** (0.26)
Output gap	0.05 (0.09)	0.07 (0.09)	0.05 (0.09)	0.07 (0.08)
Interest burden	0.81* (0.43)	0.77* (0.45)	0.79* (0.47)	0.77* (0.45)
Constant	− 5.52*** (1.46)	− 4.93*** (1.34)	− 5.36*** (1.62)	− 4.94*** (1.38)
Observations	131	131	131	131
Major debt reductions (S = 1)	31	31	31	31
McFadden R^2^	0.26	0.23	0.25	0.23
Wald χ^2^ (5) statistics	11.86	12.01	8.92	12.39
Marginal effects				
Fiscal impulse	0.04	0.04	0.04	0.04
PEXP	0.13		0.11	
REV		− 0.04		− 0.04
Trend growth	0.06	0.07	0.07	0.07
Output gap	0.01	0.01	0.01	0.01
Interest burden	0.09	0.10	0.10	0.10

Cluster robust standard errors are given in parenthesis. ***, **, *Refer to statistical significance at 1%, 5% and 10% levels, respectively

In case of dummy variables the marginal effects refer to the discrete change from 0 to 1

**Table 7:** Estimation results–omitted observations

	Threshold λ = 80		Threshold λ = 60	
	(1)	(2)	(1)	(2)
Fiscal impulse	0.65* (0.34)	0.51 (0.33)	0.65* (0.38)	0.53 (0.33)
PEXP	1.31** (0.61)		1.27** (0.61)	
REV		− 0.97 (0.74)		− 2.62 (1.72)
Trend growth	0.90** (0.36)	0.83** (0.36)	0.88** (0.34)	0.90** (0.41)
Output gap	− 0.15 (0.25)	− 0.08 (0.22)	− 0.17 (0.25)	− 0.06 (0.22)
Interest burden	0.79** (0.43)	0.81* (0.45)	0.82** (0.39)	1.07** (0.54)
Constant	− 6.72*** (1.67)	− 6.02*** (1.79)	− 6.77*** (1.64)	− 6.65*** (2.10)
Observations	66	66	66	66
Major debt reductions (S = 1)	16	16	16	16
McFadden R^2^	0.34	0.32	0.34	0.36
Wald χ^2^ (5) statistics	17.31	9.93	18.57	10.44
Marginal effects				
Fiscal impulse	0.06	0.05	0.06	0.04
PEXP	0.11		0.11	
REV		− 0.10		− 0.20
Trend growth	0.08	0.09	0.08	0.08
Output gap	− 0.01	− 0.01	− 0.02	− 0.005
Interest burden	0.07	0.08	0.07	0.07

Cluster robust standard errors are given in parenthesis. ***, **, *Refer to statistical significance at 1%, 5% and 10% levels, respectively

In case of dummy variables the marginal effects refer to the discrete change from 0 to 1

## Footnotes

1 The Czech Republic, Estonia, Hungary, Latvia, Lithuania, Poland, Slovakia and Slovenia joined in 2004. Bulgaria and Romania joined in 2007 and Croatia was the last country to join the EU in 2013 (European Commission 2015).

2 The EU15 countries include Austria, Belgium, Denmark, France, Finland, Germany, Greece, Ireland, Italy, Luxembourg, Netherlands, Portugal, Spain, Sweden and the United Kingdom. In 2020 these countries had an average gross general government debt-to-GDP ratio of 100 percent. In comparison, the average debt ratio of the eleven CEECs amounts to 54 percent of GDP in 2020.

3 This threshold is set lower than in previous studies on major debt reduction in order to take account of the generally lower debt levels in many CEECs compared to more advanced economies, such as the EU15 countries. Applying an alternative threshold of ten percentage points over five consecutive years does not change the results significantly.
